# Risk Factors and Delivery Outcomes Among Women Diagnosed With Human Papillomavirus Prior to Pregnancy Comparing High-Risk and Low-Risk Strains: A Retrospective Chart Review

**DOI:** 10.7759/cureus.106721

**Published:** 2026-04-09

**Authors:** Miranda Manzo, Emily A Hock, Natalie N Aguilar, Annie T Griepp, Jacob Surma, Chin-I Cheng, Veronika Kinaschuk

**Affiliations:** 1 College of Medicine, Central Michigan University College of Medicine, Saginaw, USA; 2 Obstetrics and Gynecology, Central Michigan University College of Medicine, Saginaw, USA; 3 Otolaryngology, Central Michigan University College of Medicine, Mount Pleasant, USA; 4 Statistics, Actuarial and Data Science, Central Michigan University, Mount Pleasant, USA

**Keywords:** awareness of human papilloma virus, high-risk pregnancy, hpv infection, human papillomavirus (hpv), maternal & neonatal outcomes

## Abstract

Introduction: Human papillomavirus (HPV) vaccination has reduced infection, but the virus remains widespread, with over 20 million new cases in the U.S. each year, with known effects on pregnancy outcomes. While prior studies have focused on infections acquired during pregnancy, less is known about how a history of HPV infection, cervical procedures, or treatment before conception may influence delivery and neonatal outcomes. Understanding these relationships could improve prenatal risk assessment and care planning.

Methods: A single-center retrospective cohort study was conducted on patients with a prior diagnosis of HPV who delivered between 2018 and 2023. The deliveries occurred among a diverse patient population at a Midwestern US hospital with approximately 3,000 births per year.

Results: Of 588 screened pregnancies, 372 met the inclusion criteria. Patients with high-risk HPV were, on average, older but otherwise demographically similar to those with other HPV strains. High-risk HPV was associated with increased delivery complications overall, including higher rates of premature rupture of membranes (OR: 0.16, 95% CI: 0.03-0.94; p=0.043), lower birth weight (p=0.006), and increased neonatal intensive care unit (NICU) admission (OR: 0.23, 95% CI: 0.05-0.97; p=0.045). A nonsignificant trend toward preterm delivery was observed (p=0.072). No differences were seen in Apgar scores or most other maternal and neonatal outcomes.

Conclusions: HPV history and strain type before conception were associated with differences in pregnancy and delivery outcomes. These findings suggest HPV history should be incorporated into prenatal risk assessment to better anticipate potential complications, though further research is needed to guide specific management strategies.

## Introduction

Human papillomavirus (HPV) infection is the most common sexually transmitted infection in the United States, with more than 42 million individuals infected at any given time. Lifetime exposure is common, with approximately 80% of sexually active individuals acquiring HPV at some point [1]. Most infections are transient and resolve spontaneously within two years; however, persistent infection can lead to the development of malignancies involving the cervix, vagina, vulva, and other parts of the body [1, 2]. Of more than 200 identified HPV types, at least 13 are oncogenic, with high-risk (HR) types 16 and 18 accounting for the majority of HPV-related cancers [2]. 

Because HPV is so common, it is important to understand how HPV infection, even in mild cases, can harm the reproductive system, leading to significant harm to reproductive health. National data from 2018 estimated that approximately 77 million people in the United States had a current HPV infection and 13 million acquired a new HPV infection that year, with those infected most often being between the ages of 15 and 24 [3]. This means women can be exposed to HPV well before they become pregnant. This raises important questions about how prior infection may influence pregnancy outcomes. 

Much of the current literature has focused on the effects of high-risk human papillomavirus (HR-HPV) infection, given the well-established role of these strains in cervical disease. Several studies have linked HR-HPV infection with preterm premature rupture of membranes, preterm birth, and placental abnormalities [4-8]. Some studies have also found associations between HR-HPV infection and hypertensive disorders of pregnancy, including preeclampsia [9]. Persistent HR-HPV infection has been connected to placental inflammation and infection, which may contribute to preterm birth and fetal growth restriction [10,11].

Other research has assessed HPV infection more broadly, without consistently distinguishing between high-risk and low-risk types. Aldhous et al. found that high-grade HPV-related cervical disease increased the risk of preterm birth, whereas low-grade cervical disease and infection without cytologic abnormality did not. This study suggests that disease severity, along with strain type, may both play roles [12]. Studies conducted in Asia and other regions have also shown that women with persistent HR-HPV infection may experience higher rates of reproductive tract inflammation and adverse pregnancy outcomes; however, these studies often grouped strain types together or used small samples [13,14]. Systematic reviews have supported a link between HPV infection during pregnancy and increased risk of spontaneous abortion, preterm birth, and preeclampsia, yet the majority of studies have not examined outcomes by HPV strain category [15].

Despite these findings, nearly all existing research has examined women with active HPV infection during pregnancy. Far less is known about whether a prior infection, after viral clearance, continues to influence pregnancy outcomes. This question is particularly important because many women are exposed to HPV at some point before or during their reproductive years. Our study was designed to address this gap by examining pregnancy and delivery outcomes in women with a documented HPV diagnosis prior to pregnancy. 

By analyzing a large and racially diverse population of urban and suburban patients, we aimed to determine whether prior HPV infection influences maternal or neonatal outcomes. This study is unique in that it isolates the effects of previous infection rather than active infection during pregnancy and directly compares the outcomes between women with prior high-risk versus low-risk HPV infection. In contrast to earlier research limited by small sample sizes or inadequate stratification, our study offers a broader and more detailed understanding of the long-term reproductive effects of HPV and its potential influence on obstetric care through a retrospective chart review. It was hypothesized that prior high-risk HPV infection is associated with increased adverse obstetric outcomes compared to low-risk strains. 

## Materials and methods

Data extraction 

This single-center retrospective cohort study was conducted at a community hospital with approximately 3,000 births per year serving a racially and geographically diverse urban and suburban population. Data were collected via retrospective electronic medical record (EMR) review. This study was approved by the Covenant Healthcare Institutional Review Board (C-24-15 HPV) with a waiver of informed consent and Health Insurance Portability and Accountability Act (HIPAA) authorization. The study included a sample of patients who were diagnosed with human papillomavirus prior to pregnancy. HPV diagnosis was identified via International Classification of Diseases 10th Revision (ICD-10) diagnostic codes documented in the EMR prior to the index pregnancy, which may reflect diagnosis by Pap smear cytology, HPV DNA co-testing, or clinical documentation. Patients included in the final data analysis were between the ages of 18 and 50 and delivered from 2018 to 2023. Eligible patient records were identified from the electronic medical record system, and each patient was assigned a study identification number. Duplicate pregnancies were removed, and confirmation of chart access was obtained prior to screening.

**Figure 1:**
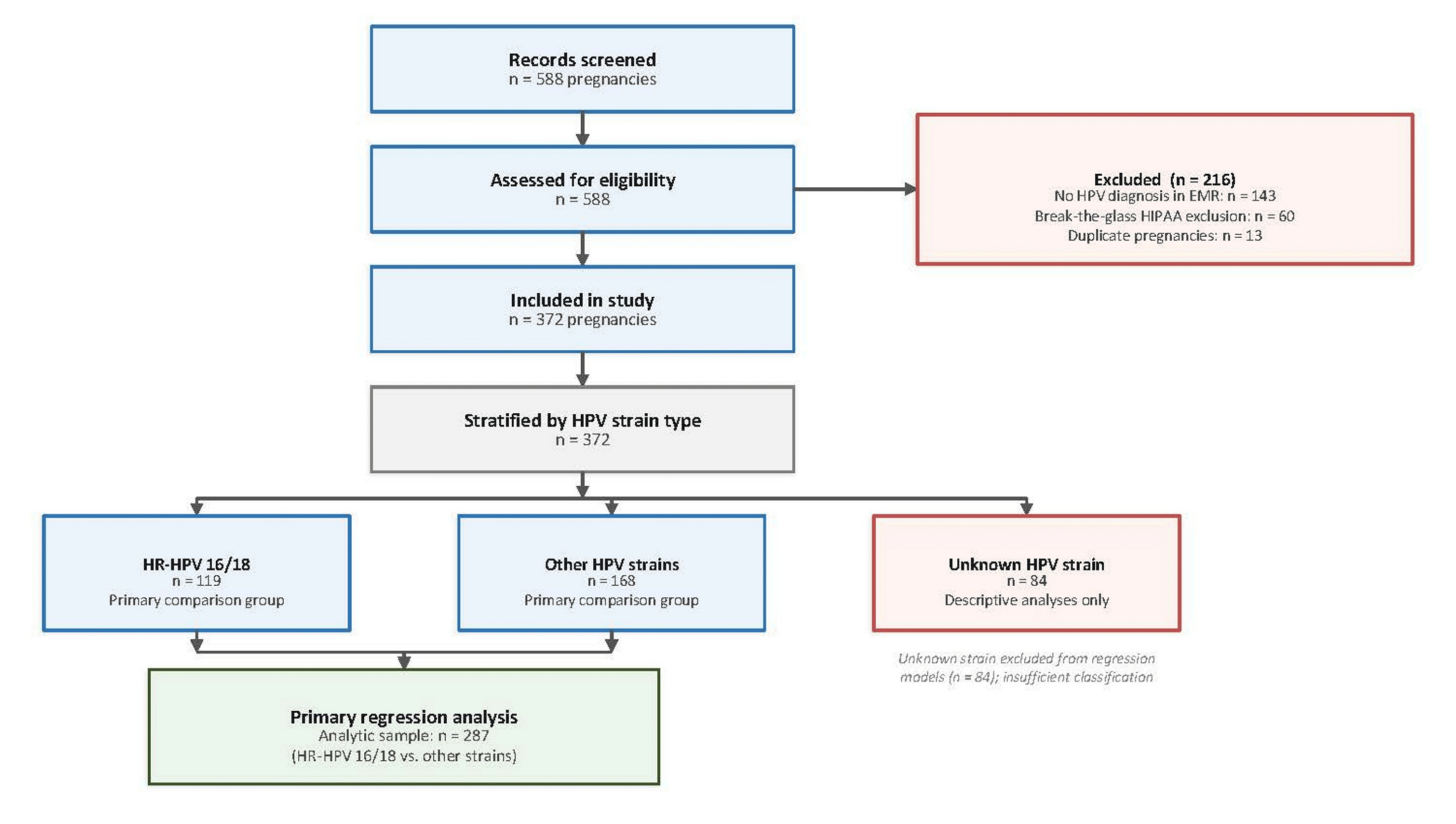
CONSORT study flow diagram. HPV: human papillomavirus, EMR: electronic medical record; HR-HPV: high-risk human papillomavirus; HIPAA: Health Insurance Portability and Accountability Act.

Exclusion criteria included a lack of a documented HPV diagnosis in the electronic medical record, restricted chart access due to “break-the-glass” HIPAA protections, and duplicate pregnancies. Primary logistic regression analyses comparing HPV strain types were limited to patients with confirmed high-risk (n=119) or other HPV strains (n=168), yielding an analytic sample of 287, as shown in Figure 1.

Patients with unknown HPV strain (n=84) were included in descriptive analyses but excluded from comparative regression models due to inability to definitively classify strain risk category. Chart abstraction was performed by four trained reviewers using a standardized data collection template with predefined variable definitions. Coding discrepancies were resolved by consensus review. Data were manually screened for included patients and coded for demographic variables, ICD-10 diagnosis codes, pregnancy complications, delivery complications, and newborn health status. Quantitative variables were coded as continuous or categorical numerical values as appropriate. HPV strain, divided into three categories of high-risk strains 16 and 18, all other strains, and unknown strain, were independent variables. Several outcome variables were studied, including delivery complications, newborn health status, and pregnancy diagnoses and complications.

Gravidity, parity, body mass index, maternal race/ethnicity, age, existing medical conditions of the mother and family, and COVID-19 status were extracted from the medical records to identify risk factors and potential confounding effects.

Additional demographics and medical information were extracted for the purposes of describing the study sample and exploring potential confounders. These variables were also evaluated as potential confounders due to their known influence on pregnancy outcomes. Substance use during pregnancy was specifically included given its established association with low birth weight, preterm delivery, and neonatal complications, as well as its prevalence within the study population. Outcome variables included ICD-10 diagnosis codes, neonatal birth weight, neonatal Apgar scores [16], neonatal health status, and maternal and neonatal complications. 

Operational definitions were established for key outcomes. Premature rupture of membranes (PROM) was defined as rupture of membranes prior to the onset of labor. Preterm delivery was defined as delivery before 37 weeks of gestation. Neonatal intensive care unit (NICU) admission was defined as documented admission of the neonate to the neonatal intensive care unit immediately following delivery according to institutional criteria.

Several potential sources of bias were considered in the design and interpretation of this study. Selection bias may have been introduced by the exclusion of 60 patients with restricted chart access, as these individuals may disproportionately represent healthcare system employees or patients who elected chart restrictions for personal privacy reasons. Measurement bias is inherent to retrospective EMR-based designs, as data accuracy depends on documentation completeness. To minimize information bias, standardized coding procedures and operational definitions were applied consistently across all chart abstractions.

Data analysis 

All analyses were performed using SAS software (version 9.4M8; SAS Institute Inc., Cary, NC, USA). Maternal descriptors were summarized using means with standard deviations for continuous variables and frequencies with percentages for categorical variables. Delivery complications, pregnancy complications, and baby status were modeled using logistic regression to estimate the odds ratios with 95% confidence intervals (CIs), with HPV strain as the primary independent variable. Neonatal complications were analyzed using proportional odds models with HPV strains. Variables with missing data were not imputed; patients with incomplete data for a given outcome were excluded from that specific analysis, resulting in variable denominators across models. All of the analytical results were considered to be significant when p-values were less than or equal to 0.05.

## Results

After data collection and exclusion criteria were applied, 588 pregnancies were screened, and a total of 372 pregnancies were included in the final analysis. Of those excluded, 143 were excluded for lack of a documented HPV diagnosis, 60 were excluded due to break-the-glass HIPAA chart protections, and 13 were duplicate pregnancies, yielding 372 pregnancies for final analysis.

Demographics

A breakdown of the demographic background of the included patients stratified by HPV strain is provided in Table 1. The average age of women with high-risk HPV strains was 32.99 years. In comparison, the average age of women with other strains was 30.64 years, with similar gravidity, parity, and gestational age at delivery among both groups. The largest ethnic group was white patients, followed by African American patients, with smaller proportions identifying as Hispanic or other ethnicities. Obesity was prevalent across all HPV groups. The mode of delivery was similar between groups, with spontaneous vaginal delivery being the most common method.

**Table 1:** Demographic and clinical characteristics by HPV strain type. HR-HPV: high-risk human papillomavirus; SD: standard deviation; BMI: body mass index. Continuous variables compared using t-tests; categorical variables compared using chi-square tests.

Characteristic	HR-HPV 16/18 (n=119)	Other strains (n=168)	Unknown (n=84)
Continuous variables, mean ± SD			
Age (years)	32.99 ± 4.29	30.64 ± 4.72	30.87 ± 4.68
Gravidity	3.42 ± 2.47	3.14 ± 1.83	3.17 ± 1.69
Parity	1.81 ± 1.50	1.57 ± 1.43	2.25 ± 1.46
Gestational age (days)	262.8 ± 31.3	266.7 ± 22.0	270.2 ± 20.1
Categorical variables, n (%)			
Ethnicity			
White	74 (62.2%)	97 (57.7%)	58 (69.1%)
African American	35 (29.4%)	62 (36.9%)	14 (16.7%)
Hispanic	5 (4.2%)	3 (1.8%)	10 (11.9%)
Other	5 (4.2%)	6 (3.6%)	2 (2.4%)
BMI category			
<25 (Normal)	16 (13.5%)	36 (21.7%)	7 (8.3%)
25-30 (Overweight)	31 (26.1%)	44 (26.5%)	20 (23.8%)
≥30 (Obese)	72 (60.5%)	86 (51.8%)	57 (67.9%)
Delivery method			
Spontaneous vaginal	69 (58.0%)	100 (59.5%)	52 (61.9%)
Cesarean section	45 (37.8%)	61 (36.3%)	30 (35.7%)
Other	5 (4.2%)	7 (4.2%)	2 (2.4%)

Pregnancy-related diagnoses

Pregnancy-related diagnoses were evaluated across ICD-10 diagnostic groups by HPV strain type, as seen in Table 2. Any maternal medical condition diagnoses overall were observed in 32.8% of patients. High-risk pregnancies accounted for 26.5%, although there were no significant differences between high-risk HPV and other HPV strains. Trends toward higher odds in patients with other HPV strains were noted for maternal medical conditions and other medical conditions. No significant differences were identified for fatal complications, history of risk factors, hypertensive disorders, diabetes, delivery complications, pregnancy symptoms, infections, membrane/fluid disorders, mental health conditions, blood/hematologic disorders, or hemorrhage/bleeding. Overall, these findings suggest that, while some trends were observed, pregnancy-related diagnoses were generally similar across HPV strain types. 

**Table 2:** ICD-10 diagnostic groups by HPV strain type. Reference group: HR-HPV types 16/18. OR > 1 indicates higher odds of condition in 'other HPV strains' compared to HR-HPV. P-values calculated using logistic regression with HR-HPV 16/18 as the reference group. †Trend (p < 0.10) ICD-10: International Classification of Diseases 10th Revision; OR: odds ratio; CI: confidence interval; HR-HPV: high-risk human papillomavirus.

ICD-10 diagnostic group	N	Events (%)	p-value	OR (95% CI)
Maternal medical conditions	287	94 (32.8%)	0.080†	1.58 (0.95-2.64)
High-risk pregnancy	287	76 (26.5%)	0.131	1.58 (0.87-2.86)
Other medical conditions	287	24 (8.4%)	0.061†	3.75 (0.94-14.98)
Fatal complications	287	27 (9.4%)	0.383	1.76 (0.49-6.29)
History of risk factors	287	40 (13.9%)	0.371	1.52 (0.61-3.76)
Hypertensive disorders	287	55 (19.2%)	0.653	1.19 (0.56-2.51)
Diabetes	287	75 (26.1%)	0.921	1.04 (0.52-2.08)
Delivery complications	287	18 (6.3%)	0.216	0.55 (0.22-1.41)
Pregnancy symptoms	287	29 (10.1%)	0.148	2.10 (0.77-5.75)
Infections	287	29 (10.1%)	0.272	1.87 (0.61-5.75)
Membrane/fluid disorders	287	16 (5.6%)	0.236	0.51 (0.16-1.56)
Mental health conditions	287	10 (3.5%)	0.409	0.50 (0.10-2.59)
Blood/hematologic disorders	287	16 (5.6%)	0.510	1.55 (0.42-5.65)
Hemorrhage/bleeding	287	8 (2.8%)	0.648	0.70 (0.16-3.17)

Maternal and obstetric outcomes

Analyses for neonatal and obstetric outcomes are shown in Tables 3-5. Logistic regression analyses identified a significant association between high-risk HPV strain and overall delivery complications. When stratified by individual delivery complication, premature rupture of membranes was significantly increased for patients with high-risk HPV strains 16 and 18. High-risk HPV strain diagnosis prior to pregnancy was significantly associated with lower neonatal birth weight. Additionally, high-risk HPV strains showed an association with premature delivery; however, this finding was not statistically significant. NICU admissions were also significantly increased in patients with high-risk HPV diagnosis prior to pregnancy. Other maternal and neonatal outcomes, including adverse obstetric outcomes and neonatal weight, were also analyzed but did not show significant differences among HPV strain groups. Substance use was also found to be significantly associated with neonatal birth weight in adjusted models (p=0.001), supporting its inclusion as an important covariate in this population. 

**Table 3:** Apgar scores and baby status by HPV strain. Reference group: HR-HPV types 16/18. Note: Full models with all covariates had convergence issues. Results shown are from reduced models (HPV variables only). Apgar scores analyzed using proportional odds models; baby status analyzed using logistic regression. HR-HPV: high-risk human papillomavirus.

Variable	Estimate/OR	Std error/95% CI	Test statistic	p-value
A. Apgar 1-minute score vs HPV strain				
Other strains vs HR-HPV 16/18	0.255	0.245	t=1.04	0.298
Unknown strain vs HR-HPV 16/18	0.374	0.298	t=1.26	0.210
B. Apgar 5-minute score vs HPV strain				
Other strains vs HR-HPV 16/18	0.086	0.322	t=0.27	0.788
Unknown strain vs HR-HPV 16/18	−0.225	0.374	t=−0.60	0.548
C. Baby status vs HPV strain				
HPV strain (overall)	-	-	χ^2^=0.29	0.865
Other strains vs HR-HPV 16/18	1.05	0.27–4.13	χ^2^=0.16	0.686
Unknown strain vs HR-HPV 16/18	0.65	0.10–4.31	χ^2^=0.28	0.599

**Table 4:** Multiple linear regression analysis for birth weight. Note: Full model controls for age, ethnicity, gravidity, parity, BMI, existing medical conditions, COVID-19 history, in vitro fertilization, blood type, gestational age, delivery method, Group B streptococcus status, substance use, preeclampsia, gestational diabetes, and human papillomavirus (HPV) procedures/treatment. *p < 0.05; **p < 0.01; † trend (p < 0.10). P-values calculated using multiple linear regression.

Variable	Estimate (g)	Std error	t-value	p-value
Full model (N=198, R²=0.77)				
HPV strain (overall effect)	-	-	F=5.32	0.006**
Strain 1 (other) vs Strain 0 (HR)	-137.8	88.9	-1.55	0.123
Strain 2 (unknown) vs Strain 0 (HR)	+256.5	134.9	1.90	0.059†
Key covariates				
Gestational age (per day)	+21.2	1.7	12.28	<0.001**
Parity	+99.5	49.8	2.00	0.048*
Substance use	+299.3	90.5	3.31	0.001**
Loop electrosurgical excision procedure (LEEP) procedure history	+125.6	130.8	0.96	0.339

**Table 5:** Individual delivery and neonatal outcomes by HPV strain type based on logistic regression models. N=287 for all analyses. Reference group: HR-HPV types 16/18. OR < 1 indicates lower odds in 'other HPV strains' compared to HR-HPV 16/18. Models are unadjusted; HPV strain entered as sole predictor. Adjusted models for selected outcomes are presented in Table 4. *p < 0.05; †trend (p < 0.10). NICU: neonatal intensive care unit; IUGR: intrauterine growth restriction; NRFHT: non-reassuring fetal heart tones; PPROM: preterm premature rupture of membranes; PROM: premature rupture of membranes; PPH: postpartum hemorrhage; ICU: intensive care unit; HR-HPV: high-risk human papillomavirus. P-values calculated using logistic regression with HR-HPV 16/18 as the reference group.

Outcome	Events (%)	OR (95% CI)	p-value
Neonatal/fetal outcomes			
NICU admission	11 (3.8%)	0.23 (0.05-0.97)	0.045*
Premature delivery	7 (2.4%)	0.19 (0.03-1.16)	0.072†
Fetal demise	5 (1.7%)	0.99 (0.19-5.16)	0.994
IUGR	3 (1.0%)	5.06 (0.26-99.97)	0.287
NRFHT	6 (2.1%)	5.06 (0.26-99.97)	0.287
Nuchal cord	14 (4.9%)	0.44 (0.15-1.33)	0.146
Fetal bradycardia	2 (0.7%)	3.59 (0.17-76.35)	0.378
Fetal respiratory distress	3 (1.0%)	0.14 (0.01-2.96)	0.206
Stillborn	1 (0.3%)	2.14 (0.09-53.66)	0.633
Decelerations	3 (1.0%)	1.19 (0.15-9.17)	0.869
Fetal opioid withdrawal	1 (0.3%)	2.14 (0.09-53.66)	0.633
Membrane and labor outcomes			
PPROM	9 (3.1%)	0.16 (0.03-0.94)	0.043*
PROM (term)	1 (0.3%)	2.14 (0.09-53.66)	0.633
Failed induction of labor	3 (1.0%)	0.42 (0.05-3.25)	0.390
Failure to descend	3 (1.0%)	0.10 (0.01-1.95)	0.128
Delivery complications			
Emergency C-section	3 (1.0%)	0.10 (0.01-1.95)	0.128
Laceration	24 (8.4%)	1.33 (0.55-3.18)	0.525
Breech presentation	12 (4.2%)	1.76 (0.49-6.29)	0.373
Shoulder dystocia	5 (1.7%)	0.71 (0.12-4.17)	0.697
Vacuum-assisted delivery	3 (1.0%)	1.67 (0.24-11.56)	0.597
Placental outcomes			
Placenta previa	4 (1.4%)	0.30 (0.04-2.06)	0.220
Placental abruption	3 (1.0%)	0.42(0.05-3.25)	0.390
Low-lying placenta	2 (0.7%)	0.71 (0.07-6.94)	0.764
Posterior placenta	2 (0.7%)	0.71 (0.07-6.94)	0.764
Cord complications			
Cord around fetal arm	2 (0.7%)	0.23 (0.01-5.87)	0.333
Cord prolapse	1 (0.3%)	2.14 (0.09-53.66)	0.633
Hemorrhage and bleeding			
Postpartum hemorrhage (PPH)	3 (1.0%)	0.42 (0.05-3.25)	0.390
Bleeding	2 (0.7%)	3.59 (0.17-76.35)	0.378
Uterine atony postpartum	2 (0.7%)	0.23 (0.01-5.87)	0.333
Severe maternal outcomes and other			
Maternal ICU admission	2 (0.7%)	0.23 (0.01-5.87)	0.333
Hysterectomy at delivery	2 (0.7%)	0.23 (0.01-5.87)	0.333
Uterine inversion	2 (0.7%)	0.71 (0.07-6.94)	0.764
Chorioamnionitis	3 (1.0%)	0.14 (0.01-2.96)	0.206
Abdominal adhesions	2 (0.7%)	0.71 (0.07-6.94)	0.764
Twins	2 (0.7%)	8.04 (0.44-148.52)	0.161

## Discussion

While it is well-established that active HPV infection during pregnancy is associated with increased adverse maternal and neonatal outcomes, there is a lack of literature and evidence on HPV infection prior to pregnancy and how this may uniquely impact outcomes [4-11]. Many women have had an HPV diagnosis prior to pregnancy and may have also undergone HPV-related cervical procedures, both of which may independently influence pregnancy outcomes. In addition to this, stratifying HPV strains helps determine if different strains of HPV are associated with these adverse effects more frequently. The present study demonstrates that a prior diagnosis of high-risk HPV, even after presumed viral clearance, is significantly associated with increased rates of premature rupture of membranes (PROM), lower birth weight, and higher NICU admissions. These findings suggest that HPV-related alterations in the reproductive tract may persist beyond active infection and continue to influence obstetric health. 

By stratifying high-risk vs. low-risk strains, this study adds granularity missing from much of the existing literature. High-risk strains present increased risk for cervical cancer as well as increased delivery complications, as shown in this study [1-3]. Several biological mechanisms may explain the outcomes identified in this study. High-risk HPV strains have been shown to induce low-grade inflammatory cytokines, which may weaken fetal membranes or disturb placental angiogenesis [6,7, 9]. Studies have shown high-risk HPV DNA in placental tissue, suggesting interference with trophoblast integrity and nutrient transport [7,15]. These mechanisms suggest potential cervical shortening or collagen remodeling predisposing women to premature rupture of membranes (PROM) [4,8-10]. Low-risk HPV strains have also demonstrated some inflammatory effects on cervical tissue; however, they are cleared more quickly and progress to malignancy far less frequently than high-risk strains. Additionally, STI co-infections such as chlamydia, gonorrhea, and bacterial vaginosis, which were not assessed in this study, represent potential residual confounders given their associations with both HPV acquisition and adverse pregnancy outcomes.

This study is consistent with prior research on HPV infection during pregnancy, further supporting that HPV infection prior to pregnancy may be associated with premature rupture of membranes, decreased birth weight, and increased NICU admission rates. Identifying women with a history of HPV infection early, notably those with high-risk strains, may help providers anticipate and prevent such complications. Many of these women diagnosed with high-risk HPV also undergo invasive procedures prior to pregnancy that affect the integrity of their cervix and may further impact pregnancy outcomes [15]. The present study suggests that women with a history of HPV who plan to become pregnant may face increased obstetric risk. Providers can help reduce this risk by encouraging HPV vaccination in adolescence, which may mitigate both infection rates and associated long-term complications. 

With knowledge about the potential adverse effects of HPV history on obstetric outcomes, providers can also work to treat other modifiable risk factors for these complications to help reduce the compounding effects of multiple risk factors. Substance use represents an important coexisting factor in this population and was included as a covariate due to its association with adverse pregnancy outcomes. In this cohort, substance use was relatively common, reflecting the demographics of an underserved and racially diverse patient population. This underscores the importance of accounting for social and behavioral determinants of health when evaluating obstetric risk. While HPV infection demonstrated independent associations with adverse outcomes, concurrent factors such as substance use may further contribute to overall risk. 

Clinically, these findings substantiate the importance of incorporating HPV history into preconception and prenatal assessments. Women with a prior diagnosis of high-risk HPV may benefit from early risk stratification and targeted surveillance. Prospective studies with larger samples are needed before specific management recommendations can be made. Moreover, the public health implications of HPV vaccination extend beyond cancer prevention; widespread vaccination may indirectly reduce obstetric complications [2]. Incorporating HPV history into prenatal risk stratification by flagging high-risk HPV in the problem list may guide earlier monitoring or referral to maternal fetal medicine, although further research is needed. 

Limitations 

While this study identifies important associations with birth outcomes, it is also important to acknowledge some potential limitations. This study's retrospective design restricts causal inference and introduces the possibility of measurement bias, as data were dependent on the accuracy and completeness of electronic medical records. Additionally, multiple statistical comparisons were performed across a wide range of maternal and neonatal outcomes, which may have increased the risk of type I error. Several outcomes had very few events (n=1-3), resulting in wide confidence intervals and unstable odds ratio estimates that should be interpreted cautiously. Findings with borderline p-values (p=0.043, p=0.045) represent fragile significance and require replication in larger cohorts before clinical conclusions are drawn. No formal a priori sample size calculation was performed, as this was a retrospective study limited by available records. A post-hoc power analysis was not conducted; however, the sample of 372 pregnancies represents a comprehensive extraction of eligible patients from the study institution over the defined period. The relatively small number of events for several outcomes limits statistical power and may reduce the reliability of individual odds ratio estimates. 

It is also important to highlight that this study did not examine STI co-infection during pregnancy to isolate potential confounding effects of other infections throughout pregnancy. Furthermore, although multiple covariates were included in adjusted models, residual confounding may still be present from unmeasured variables such as socioeconomic factors or detailed substance use patterns. 

The inclusion of an “unknown HPV strain” category may also introduce misclassification bias, as these patients could not be definitively categorized into high-risk or low-risk groups. Additionally, 60 pregnancies were excluded due to "break-the-glass" HIPAA protections, which may represent a systematically different patient population and introduce selection bias. These individuals may disproportionately represent patients employed within the healthcare system or others who have elected to restrict access to their medical records for privacy reasons, rather than representing a clinically distinct population; as such, the direction and magnitude of any resulting selection bias is uncertain. Due to restricted access, further characterization of this subgroup was not possible. 

While substance use was included as a covariate, the study did not differentiate between type, frequency, or duration of substance exposure. Larger future studies would help establish more robust effect estimates and improve generalizability. Future prospective studies incorporating HPV viral load, cervical length dynamics, and placental histopathology could help clarify the mechanistic basis and identify modifiable intervention points. 

## Conclusions

This study is among the first to evaluate pregnancy outcomes following a history of HPV infection rather than active infection. Using a large, racially diverse cohort with stratification by HPV strain type, we found that women with prior high-risk HPV infection remain at increased risk for adverse obstetric outcomes even after viral clearance. Specifically, higher rates of premature rupture of membranes, lower birth weights, and more frequent NICU admissions were observed compared to those with low-risk strains. 

These findings suggest that incorporating HPV history into preconception and prenatal care may be warranted, and that earlier monitoring for cervical changes in women with prior high-risk HPV diagnoses merits further investigation. Additionally, this research suggests that HPV vaccination may provide benefits beyond cancer prevention by potentially reducing obstetric complications. Further research is needed to determine optimal counseling, screening, and preventive care strategies for women previously infected with HPV. 
